# Mitoepigenetic Alterations in Early-Onset Parkinson’s Disease

**DOI:** 10.3390/ijms27042033

**Published:** 2026-02-21

**Authors:** Rana Abu Manneh, Paraskevi P. Chairta, Maria A. Loizidou, Maria Zanti, Andrea N. Georgiou, Kyriaki Michailidou, Christiana Demetriou, Marios Pantzaris, Eleni Zamba-Papanicolaou, Andreas Hadjisavvas

**Affiliations:** 1 Neuroepidemiology Department, The Cyprus Institute of Neurology and Genetics, Nicosia 2371, Cyprus; ranam@cing.ac.cy (R.A.M.);; 2 Cancer Genetics, Therapeutics & Ultrastructural Pathology Department, The Cyprus Institute of Neurology and Genetics, Nicosia 2371, Cyprus; 3 Biostatistics Unit, The Cyprus Institute of Neurology and Genetics, Nicosia 2371, Cyprus; 4 Department of Psychology, University of Cyprus, Nicosia 1678, Cyprus; 5 Neuroimmunology Department, The Cyprus Institute of Neurology and Genetics, Nicosia 2371, Cyprus

**Keywords:** epigenetics, mitoepigenetics, Parkinson’s disease, methylation, hydroxymethylation, single-base resolution

## Abstract

There is accumulating evidence that distinct mitochondrial DNA (mtDNA) methylation and hydroxymethylation patterns exist in Parkinson’s disease (PD). However, most studies have been limited to the investigation of specific target regions, rather than the entire mtDNA, and have been further hindered by other methodological discrepancies and the lack of non-CpG context investigation. Here, we provide a comprehensive profile of methylation and hydroxymethylation levels across the mitochondrial genome, at global and single-base resolution, in CpG and non-CpG (CHG, CHH) contexts in blood samples from early-onset PD (EOPD) patients (n = 39) and age- and sex-matched controls (n = 63). Bisulfite (BS) and oxidative-bisulfite (oxBS) conversions in parallel workflows followed by next-generation sequencing (NGS) using Illumina’s Novaseq 6000 sequencing system identified mitochondrial 5-methylcytosine (5mC) and 5-hydroxymethylcytosine (5hmC) in all contexts. Global mtDNA methylation was significantly higher in EOPD patients vs. matched controls in the CpG context (*p* = 5.63 × 10^−3^) in the BS status, and in all contexts [CpG (*p* = 2.67 × 10^−4^), CHG (*p* = 0.015), CHH (*p* = 0.012)] in the oxBS status, i.e., “true methylation”. At single-base resolution, the most statistically significant sites across the mitogenome, in the D-loop region, and CpG context, were primarily hypomethylated in EOPD patients compared to matched controls. Upon further validation, both global and base resolution mtDNA (hydroxy)methylation results could act as blood-based biomarkers for EOPD.

## 1. Introduction

Parkinson’s disease (PD) is one of the most common neurodegenerative diseases worldwide, with a pooled all-age prevalence of 1.51 cases per 1000 from 1980 to 2023 [1]. The cardinal motor symptoms of PD are bradykinesia, resting tremor, rigidity, and postural instability [2,3], whereas the associated non-motor symptoms (NMSs) include olfactory dysfunction, constipation, sleep disturbances, and depression [4]. Notably, in some patients, NMSs precede motor symptoms by decades, at a prodromal stage of the disease [5,6]. Nevertheless, the clinical diagnosis of PD requires the presence of at least two cardinal motor symptoms [6]. Since the neurodegenerative process in PD commences at an earlier presymptomatic phase, when PD is finally diagnosed, the majority of dopaminergic (DA) neurons in the substantia nigra pars compacta (SNpc) have already been lost [7,8]. Another PD hallmark is the presence of abnormal intracellular fibrillar inclusions in the central nervous system, predominantly made of Alpha-synuclein (α-syn) protein [9]. It remains to be elucidated whether α-syn aggregation is a cause or consequence of DA neuronal death observed in PD pathology.

Early-onset PD (EOPD) is a subset of PD, diagnosed between the ages of 21 and 50 inclusively [10], and accounts for 10–15% of overall cases [11]. In EOPD, bradykinesia and dystonia are more profound, while resting tremor is observed less frequently [12]. Additionally, EOPD patients have a slower disease progression [13], a slower progression of cognitive dysfunction [14], and report fewer NMS than late-onset cases [15].

Over the years, several theories for PD pathogenesis and etiology have been proposed, with mitochondrial dysfunction being one of the most prominent. Familial PD studies involving pathogenic variants in genes such as *PINK1* and *PRKN* support a direct link between mitochondrial quality control and disease risk [16,17]. In addition to genetic contributions, mitochondrial DNA (mtDNA) alterations have been reported in PD, including somatic deletions and changes in copy number in affected brain regions [18,19]. More recently, mitochondrial epigenetics (mitoepigenetics) has emerged as a possible contributor [20,21,22]. Previously, Shock et al. demonstrated that mtDNA methyltransferases (mtDNMTs) bind to the mtDNA displacement loop (D-loop) and reported that both 5-methylcytosine (5mC) and 5-hydroxymethylcytosine (5hmC) are present in mtDNA [23], suggesting that mtDNA undergoes methylation and hydroxymethylation. Further evidence showed that ageing reduces transcription of enzymes responsible for mtDNA methylation and hydroxymethylation in a mouse model [24]. Additionally, mitoepigenetic alterations have been associated with environmental toxin exposure: rotenone and paraquat exposure inhibited protein complex I of the mitochondrial oxidative phosphorylation (OXPHOS) pathway, resulting in a PD-like phenotype in rodents [25].

Human studies have described altered DNA methylation patterns in the mtDNA D-loop region and in the substantia nigra of PD patients [19,26], although the existence and functional relevance of mtDNA methylation remain debated [27,28]. To date, there is limited knowledge regarding mtDNA methylation and hydroxymethylation in PD, and the findings from published studies are controversial [18,19,26,27,28,29]. This could be attributed to variability in study design, such as differences in sample size, tissue selection, methodology, and the mtDNA region and context investigated [18,19,26,27,28,29]. Hence, given the limited knowledge in the field of mitoepigenetics and PD, further research is warranted.

Since EOPD is more strongly influenced by genetic and mitochondrial factors than late-onset disease, it represents an optimal model for investigating mitoepigenetic alterations [30,31,32]. Additionally, EOPD is frequently associated with pathogenic variants in genes involved in mitochondrial quality control and bioenergetics, particularly *PINK1* and *PRKN*, which directly regulate mitophagy and mitochondrial homeostasis [30,31,32]. Importantly, our genetic investigation of the Cypriot population identified *PINK1* and *PRKN* pathogenic variant carriers among EOPD patients, further supporting the relevance of this cohort for studying mitochondria-driven disease mechanisms [33]. The younger age of onset in EOPD minimizes confounding effects related to ageing and cumulative environmental exposure, thereby enabling a more precise assessment of primary mitoepigenetic alterations in PD pathogenesis [34,35].

Herein, we aimed to investigate whether mitoepigenetic alterations could act as diagnostic biomarkers for EOPD in the Cypriot population. DNA samples from EOPD patients and age- and sex-matched controls were used in order to perform comprehensive profiling of whole mtDNA methylation and hydroxymethylation in CpG and non-CpG sites, at global levels and single-base resolution. The “true methylation” or 5mC status of cases vs. controls, across the entire mitogenome, was determined using oxidative bisulfite (oxBS) sequencing (oxBS-Seq) and bisulfite-only (BS) sequencing (BS-Seq) in parallel workflows. This dual approach allowed the differentiation of the 5mC and 5hmC bases, i.e., methylation and hydroxymethylation, respectively.

## 2. Results

### 2.1. Sample Description

The EOPD patient cohort (n = 48) had a median age of onset of 46 years (range 23–50 years). Males had a higher frequency of EOPD compared to females (M:F ratio is 1.67:1). Family history was recorded for 18 (37.5%) patients in total; first- and second-degree relatives with PD were reported for 13 (27.1%) and 5 (10.4%) patients, respectively. In total, 47 EOPD patients and 64 age- and sex-matched controls were sequenced. One EOPD sample from the initial cohort (n = 48) was lost due to insufficient material. Analysis was performed using two different read depth (coverage) thresholds: ≥20X and ≥30X (Table 1). The samples with coverage < 20X were not included in the analyses. The sample size was determined by sample availability after applying all inclusion criteria and quality-control filters. Given the exploratory, genome-wide nature of the analyses, a formal a priori power calculation was not performed. Here we present the results at ≥30X coverage.

### 2.2. Differential Global mtDNA Methylation

**Figure 1:**
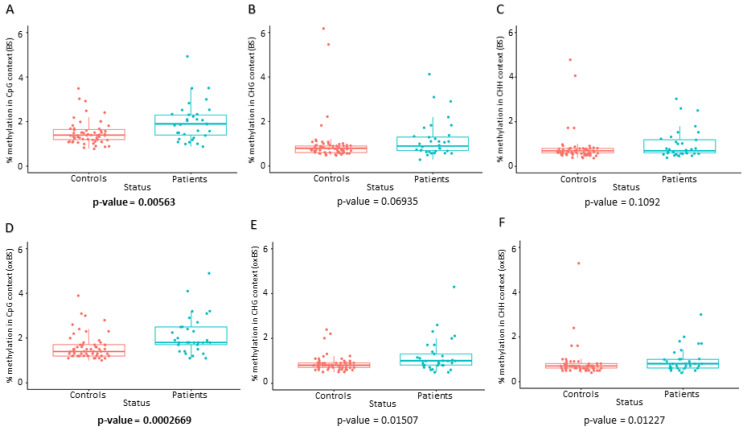
Methylation percentage boxplots. The methylation percentage in EOPD patients vs. age- and sex-matched controls in the BS (5hmC and 5mC) status in the (**A**) CpG, (**B**) CHG, and (**C**) CHH contexts, and in the oxBS (5mC) status in the (**D**) CpG, (**E**) CHG, and (**F**) CHH contexts at ≥30X coverage is illustrated. Dots and boxes in red and blue represent controls and patients, respectively. The non-parametric Wilcoxon rank sum test with continuity correction was used to compare the EOPD patients vs. age- and sex-matched controls, at ≥30X coverage. Statistically significant *p*-values after Bonferroni correction (α = 0.05/6 = 0.0083) appear in bold.

The global mtDNA methylation analysis revealed that mtDNA was significantly hypermethylated in EOPD patients (n = 33) compared to age- and sex-matched controls (n = 63) in the CpG context across both BS and oxBS statuses after Bonferroni correction (Table 2, Figure 1). CHG/CHH contexts were not significant after multiple testing correction. The data distribution of the BS and oxBS results is illustrated in boxplots (Figure 1). Since oxBS levels are nearly the same or slightly higher than BS, this implies that the 5hmC levels are negligible. The significant differences in both BS and oxBS seem to be due to changes in 5mC, not 5hmC.

### 2.3. Differential mtDNA Methylation and Hydroxymethylation at Base Resolution

The number of differentially methylated and/or hydroxymethylated bases between EOPD patients and matched controls is presented in Table 3. Since the methylation or hydroxymethylation status of a given base cannot be distinguished by BS alone, the oxidation step in the oxBS workflow allowed for the differentiation of the 5mC and 5hmC bases during the analysis. By subtracting the oxBS cytosines from the BS cytosines, the 5hmC level was derived. A substantial number of mtDNA bases were differentially methylated in the BS (5mC + 5hmC), 5mC and 5hmC statuses (Table 3).

**Figure 2:**
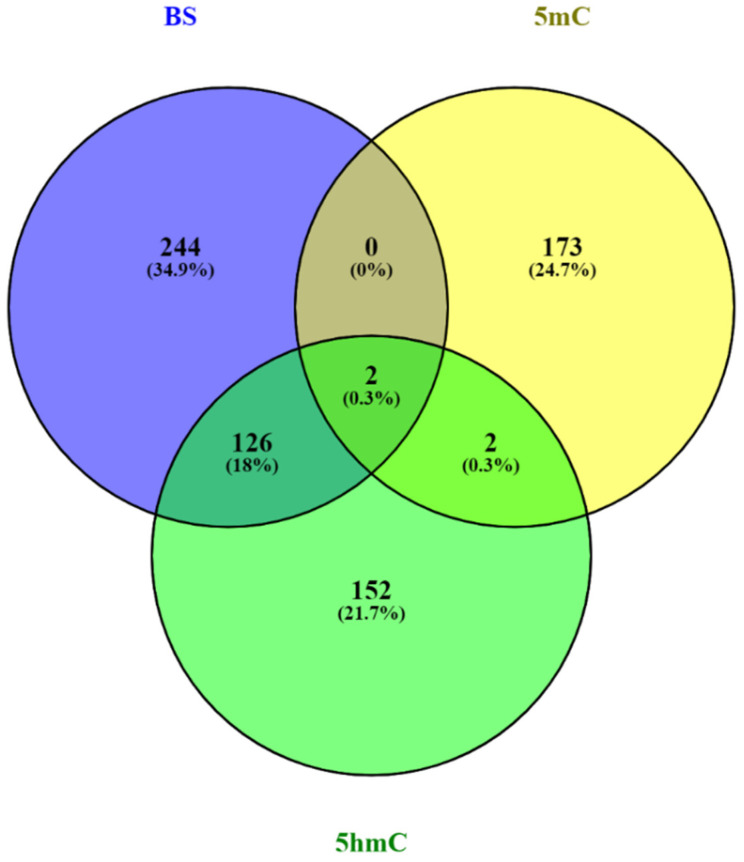
Venn diagram illustrating the number of unique and common differentially methylated bases in EOPD patients vs. matched controls across the BS (purple-blue), 5mC (yellow), and 5hmC (green) statuses. The captured bases had a fold change of ≥4 or ≤0.25 (q < 0.05).

Notably, the greatest overlap of differentially (hydroxy)methylated bases was observed between the BS (34%) and 5hmC (45%) statuses (Figure 2). The differentially methylated bases in the 5mC and 5hmC statuses seem to be exclusive, with only 2–4 bases in common between them (Figure 2). Interestingly, two bases were differentially methylated in all statuses; of which the first is located at a CHG site at position 6853 on the mtDNA light strand in the *MT-CO1* gene region, and the second at a CHH site at position 930 on the mtDNA light strand in the *MT-RNR1* gene region. Both bases were hypo(hydroxy)methylated in patients in all statuses (FC = 0.13–0.19 and FC = 0.11–0.18, respectively).

**Figure 3:**
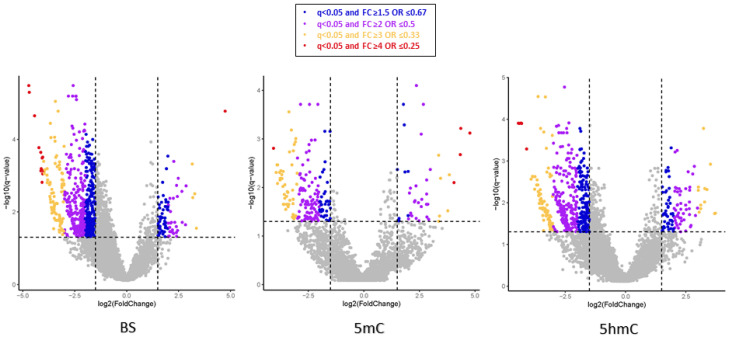
Volcano plots illustrating the distribution of differentially (hydroxy)methylated mtDNA bases in the BS, 5mC and 5hmC statuses in EOPD patients vs. matched controls. The differentially (hydroxy)methylated mtDNA bases are represented as dots, scattered based on their log2 fold change (x-axis) and −log10 q-value (y-axis; q-values calculated using the SLIM method). The dots above the horizontal threshold represent the statistically significant (q < 0.05) differentially (hydroxy)methylated bases in each status. The coloured dots represent the significantly hyper- and hypo- (hydroxy)methylated bases, with the specified fold changes (FC).

The distribution of the mtDNA bases, which were hyper(hydroxy)methylated or hypo(hydroxy)methylated in the BS, 5mC, and 5hmC statuses, is illustrated in Figure 3. Overall, the majority of bases were hypomethylated in cases vs. matched controls, indicated by a greater distribution of dots in the negative log2 FC field on the volcano plots (Figure 3).

### 2.4. The Most Differentially Methylated and Hydroxymethylated Sites in EOPD Patients vs. Matched Controls at Base Resolution

The 15 most differentially methylated sites in EOPD patients vs. age- and sex-matched controls across all contexts (CpG, CHG, CHH) in the BS, 5mC and 5hmC statuses are represented in Supplementary Tables S1, S2 and S3, respectively.

At base resolution, in the BS status, 14 out of the 15 most differentially methylated sites in EOPD patients vs. matched controls were hypo(hydroxy)methylated (Supplementary Table S1). Nevertheless, the top differentially methylated site was 26.7 times more methylated in patients than in matched controls (q = 1.68 × 10^−5^). This CHH site resides in the *MT-ND4* gene at position 10,877 on the heavy strand. Notably, 20% of the top 15 differentially methylated sites are located in the *MT-ND4* gene, and another 20% belong to sites in the *MT-CO1* gene. Only one CpG site was in the top 15 and is located in the *MT-CO3* gene.

In the 5mC status, six (40%) sites were hypermethylated compared to nine (60%) sites that were hypomethylated in patients vs. matched controls (Supplementary Table S2). Interestingly, the *MT-ND5* gene had the highest representation in the 5mC status of the differentially methylated sites, of which three of its sites were hypermethylated, and three were hypomethylated. No CpG site was represented in the top 15 differentially methylated sites in the 5mC status at base resolution.

In the 5hmC status, the profile of the sites was similar to the BS status in that only one site was hyper-hydroxymethylated in the top 15 differentially methylated sites (Supplementary Table S3). At base resolution, the top differentially methylated site was a CHH site, which was 22.2 times less hydroxymethylated in patients than in matched controls (q = 2.26 × 10^−3^). Notably, five sites were common between the 5hmC and BS statuses, namely 6904 (CHH), 6899 (CHH), 2419 (CHG), 9727 (CHH), and 11,186 (CHG).

In all three statuses, the majority of the top-most differentially methylated sites resided in mitochondrial protein-coding genes involved in the OXPHOS metabolic pathway. Additionally, the most differentially methylated sites between patients and matched controls in all three statuses were CHH sites.

With more stringent FC filters, the representation of the three contexts changes such that the majority of the C sites captured belong to the CHH context, while the CpG sites are less represented (Supplementary Figure S1). The BS and 5hmC statuses had similar patterns in terms of the context representation of their differentially methylated bases; with no FC filtering, 72–74% of the differentially methylated C sites belonged to the CHH context (Supplementary Figure S1). Furthermore, with very stringent FC filtering, i.e., FC ≥ 4 or ≤0.25, the CHH context representation increased by 11% for both BS and 5hmC statuses. Nevertheless, in the 5mC status, the CpG sites (44%) were the most prominent with no FC filtering. However, this was overturned at the FC ≥ 3 or ≤0.33 filter, where the CHH sites (41%) were more represented than the CpG sites (33%) in the 5mC status. Overall, at the FC ≥ 4 or ≤0.25 filter, the CHH context is the most represented (47–85%) across all statuses.

**Figure 4:**
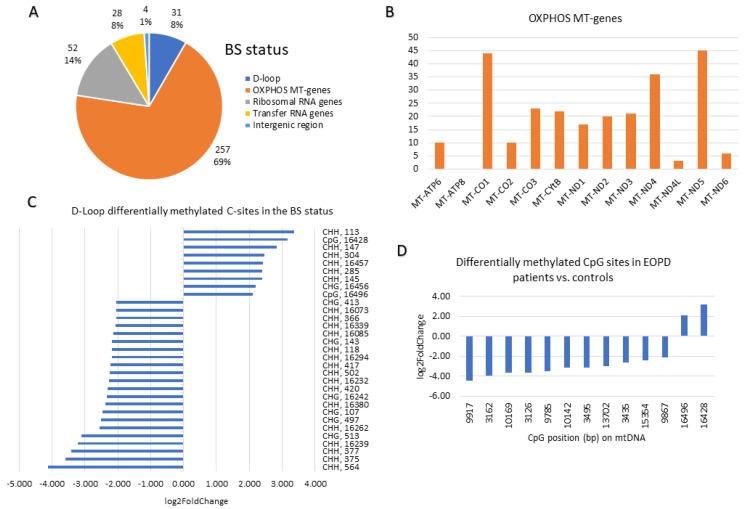
Base resolution results summary at the BS Status (FC ≥ 4 or ≤0.25): (**A**) Pie chart illustrating the number and percentage of differentially methylated sites in EOPD patients vs. age- and sex-matched controls divided according to mtDNA components. (**B**) Expansion of the OXPHOS MT-genes pie slice to indicate the genes that harbour the differentially methylated sites. (**C**) Expansion of the D-loop pie slice to represent the context and position of the hyper- and hypomethylated sites in the D-loop region. (**D**) The CpG sites in the mtDNA that were hypo- and hypermethylated in EOPD patients vs. matched controls at base resolution.

**Figure 5:**
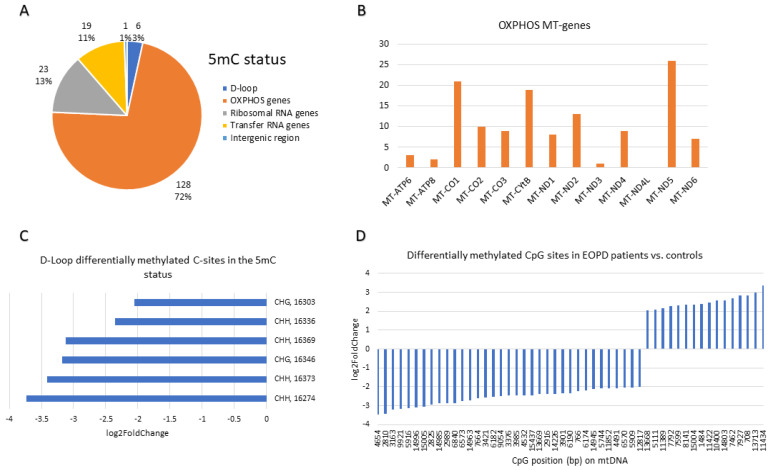
Base resolution results summary at the 5mC Status (FC ≥ 4 or ≤0.25): (**A**) Pie chart illustrating the number and percentage of differentially methylated sites in EOPD patients vs. age- and sex-matched controls divided according to mtDNA components. (**B**) Expansion of the OXPHOS MT-genes pie slice to indicate the genes that harbour the differentially methylated sites. (**C**) Expansion of the D-loop pie slice to represent the context and position of hypomethylated sites in the D-loop region. (**D**) The CpG sites in the mtDNA that were hypo- and hypermethylated in EOPD patients vs. matched controls at base resolution.

**Figure 6:**
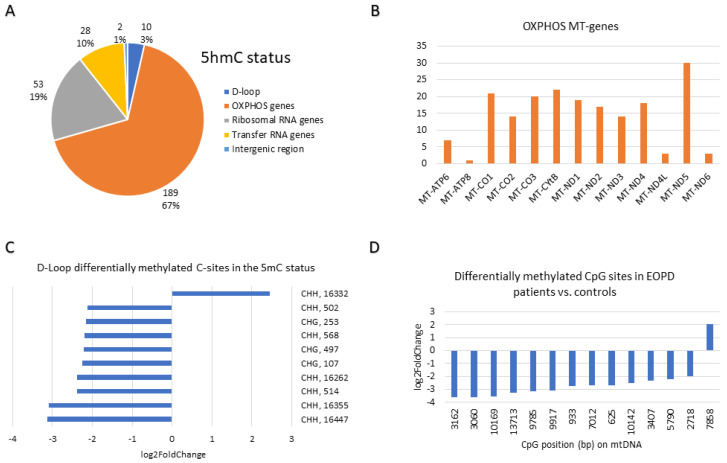
Base resolution results summary at the 5hmC Status (FC ≥ 4 or ≤0.25): (**A**) Pie chart illustrating the number and percentage of differentially methylated sites in EOPD patients vs. age- and sex-matched controls, divided according to mtDNA components. (**B**) Expansion of the OXPHOS MT-genes pie slice to indicate the genes that harbour the differentially methylated sites. (**C**) Expansion of the D-loop pie slice to represent the context and position of the hyper- and hypomethylated sites in the D-loop region. (**D**) The CpG sites in the mtDNA that were hypo- and hypermethylated in EOPD patients vs. matched controls at base resolution.

A summary of the base resolution results of the differentially methylated bases with FC ≥ 4 or ≤0.25 at the BS, 5mC and 5hmC statuses is depicted in Figure 4, Figure 5 and Figure 6, respectively. The region of the OXPHOS mitochondrial genes accounted for the majority (67–72%) of the differentially methylated sites across the three statuses (Figure 4A, Figure 5A and Figure 6A). In the BS status, the *MT-ND5* (45 sites), *MT-CO1* (44 sites), and *MT-ND4* (36 sites) genes harboured the most differentially methylated sites (Figure 4B). In the 5mC status, the *MT-ND5* (26 sites), *MT-CO1* (21 sites), and *MT-CYB* (19 sites) genes harboured the most differentially methylated sites (Figure 5B). Similar to the 5mC status, in the 5hmC status, the *MT-ND5* (30 sites), *MT-CYB* (22 sites), and *MT-CO1* (21 sites) genes contained the most differentially methylated sites (Figure 6B).

At base resolution, the D-loop region in the BS status harboured nine (29%) hyper(hydroxy)methylated (FC ≥ 4) and 22 (71%) hypo(hydroxy)methylated (FC ≤ 0.25) sites in EOPD patients vs. matched controls (Figure 4C). Conversely, in the 5mC status, all six differentially methylated sites which were found in the D-loop were hypomethylated (FC ≤ 0.25) in patients (Figure 5C), whereas, in the 5hmC status, only one hyper-hydroxymethylated site was found within the D-loop region compared with nine hypo-hydroxymethylated sites (Figure 6C). Notably, four hypo(hydroxy)methylated sites found within the D-loop region were mutual amongst the BS and 5hmC statuses, namely 502 (CHH), 497 (CHG), 107 (CHG), and 16,262 (CHH).

The mitogenome-wide CpG sites that were differentially methylated in EOPD patients vs. matched controls with FC ≥ 4 or ≤0.25 were primarily hypo(hydroxy)methylated. In the BS status, only 13 CpG sites were differentially (hydroxy)methylated in patients vs. controls, of which 11 were hypo(hydroxy)methylated and two were hyper(hydroxy)methylated (Figure 4D), whereas 35 hypomethylated CpG sites were found in the 5mC status as opposed to 16 hypermethylated sites (Figure 5D). Moreover, in the 5hmC status, only one CpG site was found to be hyper-hydroxymethylated in contrast with 13 sites which were hypo-hydroxymethylated (Figure 6D). Amongst the BS and 5hmC statuses, five hypo(hydroxy)methylated CpG sites overlapped, namely those at positions 9917, 3162, 10,169, 9785, and 10,142. Interestingly, one CpG site at position 13,713, which was more methylated in the 5mC status, was less hydroxymethylated in the 5hmC status, amongst patients.

## 3. Discussion

The published literature suggests that mtDNA and potentially mtDNA epigenetic modifications play an important role in PD pathogenesis [20]. The mtDNA is transcribed as a polycistronic mRNA, initiated by transcription factors binding to the heavy and light strand promoters (HSP1/2 and LSP, respectively) within the D-loop region [36]. Hypermethylation in the D-loop reduces mtDNA transcription [37], whereas the functional consequences of methylation in mtDNA intergenic regions remain unclear.

Compared with nuclear DNA, mtDNA is 3–10 times more susceptible to oxidative stress due to limited repair capacity [38]. Chronic exposure to environmental pollutants increases cell oxidative stress, which may sequentially damage mtDNA. Damaged mtDNA accumulates and coexists with normal mtDNA within the cell [38]. Once the ratio of damaged mtDNA to normal mtDNA exceeds a certain threshold, mitochondrial dysfunction manifests, potentially contributing to PD onset and progression [38].

Here, we present the first genome-wide mtDNA study of peripheral blood from EOPD patients and matched controls, examining whole methylation and hydroxymethylation levels across all contexts at both the global level and single-base resolution.

In this study, we used BS and oxBS sequencing to comprehensively profile mtDNA methylation and hydroxymethylation at single-base resolution, including CpG, CHG and CHH sites. The BS workflow detects both 5mC and 5hmC and therefore represents “total modified cytosines”, whereas oxBS-Seq specifically detects true 5mC (“true methylation”) by converting 5hmC to uracil (Supplementary Figure S2). By comparing BS and oxBS results, we were able to distinguish 5mC from 5hmC at both CpG and non-CpG sites, providing a high-resolution map of mitochondrial epigenetic modifications in EOPD patients and matched controls. This distinction is crucial for accurately interpreting the methylation landscape, as conventional BS-only analyses cannot differentiate between methylation and hydroxymethylation [39].

The overall global mtDNA methylation level (% methylation) was significantly higher in EOPD patients compared with matched controls. Particularly, statistically significant hypermethylation (*p* < 0.05) was observed in the CpG context across both BS and oxBS statuses after Bonferroni correction (Table 2, Figure 1). CHG/CHH contexts were not significant after multiple testing correction. Although the absolute differences in global mtDNA methylation between EOPD patients and controls were modest (~0.3–0.4%), such effect sizes are consistent with prior epigenetic studies, where small but reproducible methylation shifts can be biologically meaningful, particularly in high-copy genomes such as mtDNA [23,40]. Global mtDNA methylation provides an overall measure of methylation across the entire mitochondrial genome, while base resolution analysis gives detailed information about the methylation status of individual sites [41]. Therefore, discrepancies in coverage, leading to over- or underrepresentation of certain regions, may lead to inaccuracies in determining the actual global mtDNA methylation.

Several studies investigated genome-wide methylation levels in PD through epigenome-wide association studies [27,29]. Chuang et al. used Illumina’s HumanMethylation450 BeadChip (San Diego, CA, USA) to detect genome-wide differentially methylated CpG sites in a large cohort of PD patients and controls [29]; however, it is unclear whether mtDNA CpG sites were accounted for, as the authors did not comment on the mtDNA CpG methylation status. Similarly, a study by Gonzalez-Latapi et al., which used Illumina‘s Infinium MethylationEPIC array, did not clarify if mtDNA CpG sites were included on the BeadChip, and none were reported to be altered in patients vs. controls [42]. On the other hand, Lüth et al. used genome-wide Nanopore sequencing technology to investigate mtDNA CpG methylation and identified significantly lower overall CpG methylation in five Parkin-PD patients compared with three controls [27]. Nevertheless, their study was limited to a very small sample size, a lack of non-CpG context consideration, and a lack of distinction between methylated and hydroxymethylated CpG sites [27].

In this study, methylation at base resolution in all contexts in the oxBS status, namely CpG, CHG and CHH, was identified. This indicates that “true methylation”, i.e., the conversion of C to 5mC, indeed occurs at CpG and non-CpG (CHG, CHH) sites in the mtDNA, consistent with the findings of other studies [40,43,44]. It is of importance that the present study specifically distinguishes the presence of 5mC in CpG and non-CpG sites across the mtDNA. The existence of extensive non-CpG methylation in human mtDNA has been controversial. While Patil and colleagues showed evidence of extensive mtDNA methylation in CHG and CHH contexts [44], their work was refuted by Guitton et al. on the basis of methodological and technical weaknesses [45]. Further, one of their arguments against Patil et al. was that the methylation proportion cut-off was oversimplified and did not reflect the reality that every C can have a methylation proportion from 0% to 100%. On the contrary, in this study, the actual values of the methylation proportion per C were used for the differential methylation analysis. Hence, the methylation proportion per C site was accounted for across all contexts.

We applied standard filters to identify differentially (hydroxy)methylated CpG and non-CpG loci between cases and controls across the entire mitogenome at single-base resolution. Notably, the majority of the differentially (hydroxy)methylated sites were hypo(hydroxy)methylated in EOPD patients vs. matched controls in all three statuses, as reflected also in the top 15 most differentially (hydroxy)methylated sites. This is in contrast to the global mtDNA % methylation results, which demonstrated that EOPD patients were significantly hypermethylated at the global mtDNA methylation level in comparison with matched controls.

The topmost differentially methylated site between patients and matched controls belonged to the CHH context. Nonetheless, the topmost CHH site in each status was exclusive. Additionally, at the FC ≥ 4 or ≤0.25 filter, the CHH context represented the majority of the differentially methylated sites between cases and controls. It is noted that the majority of studies in the field of mtDNA methylation in PD investigated CpG sites [18,19,26,27,29]. Therefore, the CpG context needed to be considered for comparison with the results of the other studies.

Hence, examining the CpG context in the BS status revealed 11 (85%) significantly hypomethylated loci (FC ≤ 0.25, q < 0.05) in EOPD patients vs. matched controls, in contrast with 2 (15%) hypermethylated loci (FC ≥ 4, q < 0.05). Similarly, in the 5hmC status, 1 (7%) CpG site was hypermethylated, and 13 (93%) CpG sites were hypomethylated in cases vs. controls, of which 5/13 and 1/13 were shared amongst the BS and 5mC statuses, respectively. Interestingly, in the 5mC status, the CpG sites lost their representation with more stringent FC filters and were not represented at all in the 15 most differentially methylated bases. However, when exclusively examining the CpG context, 35 (69%) significantly hypomethylated (FC ≤ 0.25, q < 0.05) sites in EOPD patients vs. matched controls were reported at the mitogenome-wide level, compared with 16 (31%) hypermethylated sites (FC ≥ 4, q < 0.05).

To date, three studies investigated (hydroxy)methylation in specific mtDNA regions amongst PD patients and controls [18,19,26]. Namely, Blanch et al. studied methylation and hydroxymethylation in the D-loop region, *MT-ND1*, and *MT-ND6* genes in mtDNA from substantia nigra (SN) tissue of 10 PD patients and 10 controls [26]. They detected D-loop hypomethylation (5mC) in PD patients, with no differences in the hydroxymethylation (5hmC) status in the three aforementioned regions among the groups [26]. Even though the source of mtDNA in our study was peripheral blood, as opposed to SN tissue in the study by Blanch et al. [26], we also identified D-loop hypomethylation in patients at base-resolution. Specifically, in the 5mC status, the differentially methylated sites in the D-loop region (n = 6) were exclusively hypomethylated (100%) in EOPD vs. controls. Contrary to Blanch et al. [26], we identified hydroxymethylation differences in cases vs. controls. Namely, the 5hmC status harboured 10 differentially methylated bases in the D-loop region, of which nine (90%) were hypomethylated (FC ≤ 0.25, q < 0.05) and one was hypermethylated (FC ≥ 4, q < 0.05). Two recent studies that examined mtDNA methylation in the CpG context in four mitochondrial genes [18] and in the D-loop region [19] found no differences between PD patients and controls [18,19]. Even though both studies analyzed DNA isolated from peripheral blood, as in our study [18,19], they did not investigate non-CpG (hydroxy)methylation, which could explain the discrepancies with the current study. Stoccoro et al. did not detect any CpG methylation differences in the D-loop region in PD patients [19]. Nevertheless, the methylation (5mC) differences detected in this study in the D-loop region were in non-CpG sites. Sharma et al. did not identify methylation differences in the CpG context in *MT-CO1*, *MT-CO2*, and *MT-CO3* amongst PD patients [18]. The present study identified a hypomethylated CHG site in EOPD patients vs. matched controls, at position 6853 on the mtDNA light strand (-) in *MT-CO1*, in all statuses, amid many other significantly altered non-CpG sites.

It was previously reported that hypomethylation in the D-loop region is associated with an increase in mtDNA copy number in patients, indicating an increase in the replication rates of the mitogenome [46]. Subsequently, it was suggested that increasing mtDNA may compensate for the mitochondrial dysfunction in specific patients [46], potentially increasing transcription rates, which may aid in ATP production and homeostasis recovery efforts. Furthermore, data from several studies suggest that D-loop region methylation levels may act as progression biomarkers for the process of neurodegeneration [20]. While our study used peripheral blood rather than neuronal tissue, previous work has shown that DNA methylation patterns can be detectable in both brain and blood in Parkinson’s disease and can distinguish patients from controls, supporting the utility of blood-based methylation profiles as surrogate non-invasive biomarkers for neurodegenerative processes [47,48]. However, further work is needed to clarify how well peripheral mitoepigenetics reflect brain mitochondrial changes.

When stratifying the most differentially methylated bases (FC ≥ 4 or ≤0.25) based on region, 67–72% resided in mitochondrial OXPHOS genes, which encode the protein subunits of the OXPHOS machinery complexes. The most prominent mitochondrial gene that was significantly differentially methylated was *MT-ND5* across all statuses. The *MT-ND5* gene encodes one of the seven subunits transcribed from the mtDNA to form the complex I enzyme (NADH-ubiquinone oxidoreductase), the largest of the OXPHOS complexes. Although the majority of differentially (hydroxy)methylated sites identified in this study were located within mitochondrial OXPHOS genes, including MT-ND5, the present work does not directly assess mitochondrial transcriptional output, respiratory chain activity, or cellular bioenergetics. Therefore, a direct functional link between the observed mtDNA (hydroxy)methylation changes and bioenergetic deficits in EOPD cannot be established from these data alone. Nevertheless, given the central role of MT-ND5 and other OXPHOS genes in Complex I assembly and function—one of the most consistently impaired mitochondrial complexes in PD—the identified epigenetic alterations are plausible and may contribute to mitochondrial dysfunction by modulating mtDNA transcription or replication. Functional studies integrating mtDNA epigenetic profiling with measures of mitochondrial gene expression and respiratory capacity will be required to validate this hypothesis.

Overall, it was challenging to compare the results of this study to other published studies in the field [18,19,26,27,29], mainly due to dissimilarities and discrepancies in the methodologies, i.e., source of mtDNA, sample size, technologies used, and mtDNA region investigated. Nevertheless, our study was the first to comprehensively evaluate mtDNA methylation and hydroxymethylation levels in EOPD and matched controls. While our EOPD cohort was the largest to be investigated in this context to date, the absolute sample size remains modest due to disease rarity. Another advantage of our study is that we examined 5mC status, which represents true methylation. The BS status includes both methylated and hydroxymethylated cytosines, and seems to be more representative of the 5hmC rather than the 5mC status.

The elucidation of the molecular mechanisms of PD pathogenesis, and in particular, the epigenetic mechanisms by which mtDNA methylation and hydroxymethylation affect disease onset and progression, holds great promise for the future of PD research. Our study contributes to the advancement of this burgeoning field since we provide evidence that altered mtDNA methylation and hydroxymethylation levels at global and base resolution detected in the peripheral blood may serve as epigenetic biomarkers for EOPD.

## 4. Materials and Methods

### 4.1. Study Population

The patient cohort is described in detail in Abu Manneh et al. [33]. In summary, EOPD patients of Greek-Cypriot origin were recruited from the neurology clinics of the Cyprus Institute of Neurology and Genetics (CING) in Nicosia, Cyprus. All participating patients were evaluated by CING neurologists and fulfilled the inclusion criteria, which were (i) clinical diagnosis of PD, and (ii) age of onset between 21 and 50 years. Patients with additional neurological symptoms were excluded from the study. EOPD patients were recruited between 2014 and 2021, when CING was the only tertiary referral centre for genetic and neurological disorders in Cyprus, representing the majority of EOPD patients in Cyprus at that time. Age, gender, and ethnically matched healthy controls were also recruited using random cluster sampling from the general population. The study was approved by the Cyprus National Bioethics Committee and conducted in accordance with the 1964 Declaration of Helsinki. Informed consent was obtained from all participants.

Peripheral blood was collected in EDTA-containing tubes (two 9 mL tubes per participant). Genomic DNA was extracted from peripheral blood lymphocytes using a standard phenol–chloroform extraction protocol. Briefly, red blood cells were removed by repeated lysis and centrifugation, followed by white blood cell lysis and protein digestion with Proteinase K. DNA was subsequently purified by sequential phenol–chloroform and chloroform extractions, ethanol-precipitated, and resuspended in HPLC-grade water. DNA concentration and purity were assessed using a NanoDrop^®^ ND-1000 spectrophotometer (Thermo Fisher Scientific, Waltham, MA, USA). Subsequently, mtDNA methylation was assessed using total genomic DNA. This approach is commonly used in mtDNA methylation studies, as the high copy number of mitochondrial genomes per cell allows reliable detection of mtDNA-derived signals from total DNA preparations [23,40].

### 4.2. NGS Library Preparation

Library preparation of 1 μg input DNA was performed with the “Ovation^®^ Ultralow Methyl-Seq” kit (NuGEN^®^ Technologies, Redwood City, CA, USA; TECAN Genomics, Männedorf, Switzerland).

Commercially available 897 bp synthetic DNA (D4505, Zymo Research, Irvine, CA, USA) was used as a control, where all cytosines were either unmodified or modified to 5hmC. 0.10% of the synthetic DNA was pooled with 1 μg DNA of each sample prior to BS/oxBS conversion to verify conversion efficiency and confirm reliable 5mc/5hmC detection across all libraries. The NGS library preparation protocol was performed according to the manufacturer’s instructions. This kit is compatible with Illumina sequencing platforms and contains 96 (Part Nos. 9513) unique barcoded adaptors for multiplex sequencing. All sequencing analysis runs were performed on a NovaSeq 6000 system (Illumina).

In summary, the Covaris Adaptive Focused Acoustic method was applied to fragment the DNA samples to a 200 base-pair length using the M220 Focused-ultrasonicator (Covaris^®^, Woburn, MA, USA). After the fragmentation step, each sample was aliquoted into 2 new tubes for parallel processing via the BS and oxBS workflows. Consequently, this allowed for the quantification of the 5mC and 5hmC content (Supplementary Figure S2). End repair of the fragmented samples generated blunt ends that were utilized for adaptor ligation. A 96-plex adaptor plate, including unique 8-base barcodes for each adaptor mix, was used. Final repair, oxBS conversion, and PCR steps followed.

Magnetic beads were used at specified DNA purification steps throughout the aforementioned protocol. The general process included the binding of the DNA to the beads, the magnetic separation of the beads from the supernatant, washing steps to discard contaminants, and finally, an elution step to release the DNA from the magnetic beads back into the solution.

### 4.3. Quantitative Assessment of the NGS Library

The NGS library was quantified using the Qubit™ 3 Fluorometer instrument, dsDNA broad range (BR) or high sensitivity (HS) standards, buffers and dye (Thermo Fisher Scientific, Waltham, MA, USA).

### 4.4. Qualitative Assessment of the NGS Library

Quality assessment of the NGS Library was performed using the TapeStation system, High Sensitivity D1000 ScreenTape and Sample Buffer (Agilent, Santa Clara, CA, USA).

### 4.5. NGS Run

Ninety-six samples were run at a time. Following the last step of the NGS library preparation, samples were normalized to 2nM. Then, 10 μL of each sample was added to make the library pool.

The following formula was used to calculate the required concentration of the library pool for a 2nM final molarity, based on the average library size obtained from the qualitative assessment:

(1) $$\frac{\left(concentration\;\mathrm{in}\;\mathrm{ng}/\mu L\right)}{(660\;g/\mathrm{mol}\;\times \;average\;library\;size)\;}\;\times \;10^{6}=concentration\;\mathrm{in}\;\mathrm{nM}$$

The library pool was then measured using the HS Qubit™ method and normalized to reach a 2nM final molarity.

The standard loading protocol from the Novaseq 6000 Denature and Dilute Libraries Guide (Document # 1000000106351 v03, Illumina, San Diego, CA, USA) was followed for the next steps. In detail, a 5% PhiX control was added to 99 μL of the library pool. Then, 25 μL of 0.2 NaOH was added, and the tube was vortexed briefly. This was followed by a quick spin and incubation at RT for 8 min to allow for the denaturation of the library pool. Subsequently, 25 μL Tris-HCl (400 mM, pH 8.0) was added to neutralize it, followed by a quick vortex and spin-down step. The full volume of the denatured library was transferred to the library tube provided with the NovaSeq 6000 Reagent Kit (Illumina, San Diego, CA, USA). The library tube was then loaded into the cluster cartridge the run was set up. Sequencing was performed on the Novaseq 6000 platform (Illumina, San Diego, CA, USA).

### 4.6. Bioinformatics and Statistical Analysis

#### 4.6.1. Zymo Control Analysis

Zymo quality control analysis was performed using the cegx_bsExpress programme (https://github.com/cegx-ds/cegx_bsExpress, v0.5cegx, accessed on 8 February 2023). The following tools were implemented for the analysis; Bismark v0.14.0, Perl v5.22.1, R v3.3.2, Python v2.7.12, Java v1.8.0_92, bedtools v2.26.0, samtools v1.4, Bowtie2 v2.2.9. The following script was used for the zymo control analysis; bsExpress -i <fastq file> -r <zymo fasta file> --outdir <output directory> --skip_trim -p <prefix> --skip_clip --bismark_path <bismark>.

#### 4.6.2. Methylation Analysis

Adapter and quality trimming were performed for both BS-Seq and oxBS-Seq reads using the Cutadapt tool (v1.9, https://cutadapt.readthedocs.io/en/stable/, accessed on 8 February 2023). Subsequently, trimmed reads were aligned to the human mitochondrial genome (mitogenome) (NC_012920.1) using the Bismark algorithm (v0.23, https://www.bioinformatics.babraham.ac.uk/projects/bismark/, accessed on 8 February 2023). Next, PCR duplicates were removed using the deduplicate_bismark tool. Finally, methylation calling was performed using the bismark_methylation_extractor module of Bismark. Base-pair level differential methylation analysis was implemented using the R package methylKit (v1.12, https://bioconductor.org/packages/release/bioc/html/methylKit.html, accessed on 8 February 2023). The minimum read coverage was set to 30X. Pair-wise comparisons were performed in methylKit using logistic regression. Before differential methylation analysis, sequential steps of coverage normalization and 5mC and 5hmC methylation adjustment were performed using methylKit functions. The percentage of (%) methylation in each of the groups was compared using the non-parametric Wilcoxon rank sum test. Prior to group comparisons, the distribution of methylation levels was assessed for normality using the Shapiro–Wilk test, and homogeneity of variances between patients and controls was evaluated using Levene’s test with median centering. Bonferroni correction was applied to account for multiple testing.

#### 4.6.3. Base Resolution Analysis

To perform base resolution analysis, several filtering and normalization steps were implemented. First, the function methylKit::filterByCoverage(BS, lo.count = 10, lo.perc = NULL, hi.count = NULL, hi.perc = 99.9) was used to exclude bases with coverage < 30X and bases with coverage higher than this percentile, removing outliers with extremely extensive coverage compared to the overall set. Next, methylKit::normalizeCoverage(filtered.BS, method = “median”) was applied to normalize coverage values between samples using a scaling factor derived from differences between the median of coverage distributions. The function methylKit::unite(norm_true_BS, min.per.group = 5L) was used to exclude bases covered by fewer than five samples. Finally, methylKit::calculateDiffMeth(uniteBS, covariates = covs) was performed to calculate differential methylation between the groups, adjusting for age and sex as covariates. *p*-values from this analysis were adjusted to q-values using the SLIM method [49], which estimates the proportion of true null hypotheses while accounting for dependence structures across genomic positions.

#### 4.6.4. Data Visualization

Venn diagrams (https://bioinfogp.cnb.csic.es/tools/venny/, accessed on 12 April 2023) were used to illustrate the unique and common differentially methylated bases amongst the different statuses (BS, 5mC and 5mhC) with a q-value (q) < 0.05, i.e., the adjusted *p*-value, and fold change (FC) ≥ 4 or ≤0.25.

## Figures and Tables

**Table 1:** Number of samples collected and used for analysis in this study.

	No. of Samples Sequenced	No. of Samples with ≥10X Coverage	No. of Samples with ≥20X Coverage	No. of Samples with ≥30X Coverage
EOPD patients	47	46	39	33
Age- & sex-matched controls	64	64	63	63
EOPD, early-onset Parkinson’s disease.				

**Table 2:** Methylation percentage in BS and oxBS statuses across all contexts.

	**% Methylation in BS (5mC & 5hmC) Status**		
	*CpG context*	*CHG context*	*CHH context*
Controls (mean ± SD)	1.65 ± 1.04	0.97 ± 0.93	0.80 ± 0.71
Patients (mean ± SD)	1.98 ± 0.86	1.20 ± 0.83	0.99 ± 0.65
*p*-value ^1^	**0.00563**	0.06935	0.1092
	**% Methylation in oxBS (5mC) status**		
	*CpG context*	*CHG context*	*CHH context*
Controls (mean ± SD)	1.70 ± 1.00	0.97 ± 0.82	0.80 ± 0.65
Patients (mean ± SD)	2.12 ± 0.85	1.20 ± 0.76	0.97 ± 0.54
*p*-value ^1^	**0.0002669**	0.01507	0.01227

^1^ The non-parametric Wilcoxon rank sum test with continuity correction was used to compare the EOPD patients vs. age- and sex-matched controls, at ≥30X coverage. Statistically significant *p*-values after Bonferroni correction (α = 0.05/6 = 0.0083) appear in bold.

**Table 3:** Differentially methylated and/or hydroxymethylated bases at base resolution.

Criteria	Status		
	BS	5mC	5hmC
no FC filter	1268	267	694
FC ≥ 1.5 or ≤0.67	1265	267	679
FC ≥ 2 or ≤0.50	1107	261	627
FC ≥ 3 or ≤0.33	622	216	413
FC ≥ 4 or ≤0.25	372	177	282

FC, fold change; BS, bisulfite; 5mC, 5-methylcytosine; 5hmC, 5-hydroxymethylcytosine. Values are presented as numbers (n) of differentially methylated and/or hydroxymethylated bases between EOPD patients and age- and sex-matched controls in the BS, 5mC, and 5hmC statuses with different fold change filters (q < 0.05).

## Data Availability

The original contributions presented in this study are included in the article/Supplementary Materials. Further inquiries can be directed to the corresponding authors.

## Supplementary Materials

The following supporting information can be downloaded at: https://www.mdpi.com/article/10.3390/ijms27042033/s1.
