# Supplementary material for: Mitoepigenetic Alterations in Early-Onset Parkinson’s Disease
Source: Int J Mol Sci. 2026 Feb 21;27(4):2033. doi: 10.3390/ijms27042033 (PMC12941377; doi:10.3390/ijms27042033)
Supplement: Supplementary file 1 [file ijms-27-02033-s001.zip › ijms-4113638-supplementary.pdf]

Supplementary Table S1 |The 15 most differentially methylated sites in EOPD patients vs. age- and sex-matched controls across all contexts (CpG, CHG, CHH) in the BS status at ≥30X.

Context	Position	Strand	p-value	q-value [1]	Controls [2]	EOPD Cases [2]	Δ Methylation [3]	Fold Change	Log2 Fold Change	Gene/ rRNA/ tRNA [4]	Protein Accession
CHH	10877	+	6.71E-08	1.68E-05	0.04	1.17	1.13	26.73	4.74	*MT-ND4*	YP_003024035.1
CHH	6904	-	1.65E-09	3.32E-06	1.26	0.05	-1.21	0.04	-4.71	*MT-CO1*	YP_003024028.1
CHH	6899	-	3.87E-09	5.09E-06	1.24	0.05	-1.19	0.04	-4.69	*MT-CO1*	YP_003024028.1
CpG	9917	+	3.38E-08	2.25E-05	1.08	0.05	-1.03	0.05	-4.43	*MT-CO3*	YP_003024032.1
CHH	1192	+	1.74E-06	1.70E-04	1.09	0.06	-1.03	0.05	-4.22	*MT-RNR1*	_
CHH	7260	+	2.69E-06	2.29E-04	0.90	0.05	-0.85	0.06	-4.13	*MT-CO1*	YP_003024028.1
CHH	564	-	2.10E-05	7.39E-04	1.68	0.10	-1.58	0.06	-4.13	D-loop	_
CHH	9727	+	1.47E-05	6.46E-04	1.13	0.07	-1.06	0.06	-4.11	*MT-CO3*	YP_003024032.1
CHH	1574	-	6.30E-05	1.53E-03	0.88	0.05	-0.83	0.06	-4.07	*MT-RNR1*	_
CHG	2419	+	6.10E-06	3.27E-04	0.81	0.05	-0.76	0.06	-4.07	*MT-RNR2*	_
CHH	10145	+	2.88E-05	8.96E-04	1.08	0.07	-1.01	0.06	-4.04	*MT-ND3*	YP_003024033.1
CHG	5847	+	5.31E-06	3.09E-04	0.94	0.06	-0.88	0.06	-4.04	*MT-TY*	_
CHH	4478	+	2.05E-05	7.28E-04	0.95	0.06	-0.89	0.06	-4.02	*MT-ND2*	YP_003024027.1
CHG	11186	+	1.22E-05	4.05E-04	1.14	0.07	-1.07	0.06	-3.99	*MT-ND4*	YP_003024035.1
CHH	11185	+	2.77E-05	8.75E-04	1.15	0.07	-1.08	0.06	-3.97	*MT-ND4*	YP_003024035.1

+, heavy strand; -, light strand; rRNA, ribosomal RNA; tRNA, transfer RNA; [1] adjusted p-value, [2] mean methylation percentage, [3] difference in methylation percentages between patients and controls (patients - controls), [4] elements in the exact position of the methylation site.

Supplementary Table S2 | The 15 most differentially methylated sites in EOPD patients vs. age- and sex-matched controls across all contexts (CpG, CHG, CHH) in the 5mC status at ≥30X.

Context	Position	Strand	p-value	q-value [1]	Controls [2]	EOPD Cases [2]	Δ Methylation [3]	Fold Change	Log2 Fold Change	Gene/ rRNA/ tRNA [4]	Protein Accession
CHH	4309	-	8.46E-07	7.60E-04	0.024	0.664	0.640	27.3	4.8	*MT-TI*	_
CHH	14471	+	4.07E-07	6.10E-04	0.035	0.722	0.687	20.5	4.4	*MT-ND6 **	YP_003024037.1
CHH	7467	+	6.59E-06	2.12E-03	0.030	0.605	0.575	20.2	4.3	*MT-TS1*	_
CHH	13368	-	3.14E-06	1.57E-03	0.644	0.038	-0.606	0.1	-4.1	*MT-ND5*	YP_003024036.1
CHH	13720	+	1.59E-04	7.97E-03	0.050	0.830	0.780	16.6	4.1	*MT-ND5*	YP_003024036.1
CHH	13477	-	5.98E-05	4.93E-03	0.642	0.042	-0.601	0.1	-3.9	*MT-ND5*	YP_003024036.1
CHH	12070	-	2.56E-05	3.44E-03	0.609	0.041	-0.569	0.1	-3.9	*MT-ND4*	YP_003024035.1
CHH	11559	-	1.77E-04	8.30E-03	0.659	0.045	-0.615	0.1	-3.9	*MT-ND4*	YP_003024035.1
CHH	5293	-	2.42E-05	3.44E-03	0.692	0.047	-0.646	0.1	-3.9	*MT-ND2*	YP_003024027.1
CHG	12682	+	2.72E-04	5.50E-03	0.029	0.411	0.382	14.4	3.8	*MT-ND5*	YP_003024036.1
CHH	7075	-	5.95E-05	4.93E-03	0.630	0.045	-0.585	0.1	-3.8	*MT-CO1*	YP_003024028.1
CHH	12670	+	1.81E-03	3.03E-02	0.031	0.421	0.390	13.7	3.8	*MT-ND5*	YP_003024036.1
CHH	13634	-	1.22E-04	7.06E-03	0.763	0.056	-0.707	0.1	-3.8	*MT-ND5*	YP_003024036.1
CHH	12168	-	1.01E-04	6.50E-03	0.489	0.036	-0.453	0.1	-3.8	*MT-TH*	_
CHH	16274	-	4.94E-05	4.53E-03	0.836	0.063	-0.774	0.1	-3.7	D-loop	_

+, heavy strand; -, light strand; rRNA, ribosomal RNA; tRNA, transfer RNA; [1] adjusted p-value, [2] mean methylation percentage, [3] difference in methylation percentages between patients and controls (patients - controls), [4] elements in the exact position of the methylation site, * gene resides on light strand.

Supplementary Table S3 | The 15 most differentially methylated sites in EOPD patients vs. age- and sex-matched controls across all contexts (CpG, CHG, CHH) in the 5hmC status at ≥30X.

Context	Position	Strand	p-value	q-value [1]	Controls [2]	EOPD Cases [2]	Δ Methylation [3]	Fold Change	Log2 Fold Change	Gene/ rRNA/ tRNA [4]	Protein Accession
CHH	12345	-	3.59E-05	2.26E-03	1.11	0.05	-1.06	0.04	-4.48	*MT-ND5*	YP_003024036.1
CHH	6132	-	2.25E-05	2.14E-03	1.16	0.05	-1.11	0.05	-4.42	*MT-CO1*	YP_003024028.1
CHH	6899	-	8.53E-05	3.68E-03	1.13	0.05	-1.08	0.05	-4.40	*MT-CO1*	YP_003024028.1
CHH	6904	-	3.40E-05	2.24E-03	1.13	0.05	-1.08	0.05	-4.39	*MT-CO1*	YP_003024028.1
CHH	12606	+	2.97E-08	4.24E-05	1.31	0.06	-1.24	0.05	-4.34	*MT-ND5*	YP_003024036.1
CHH	4149	+	3.96E-03	3.30E-02	1.14	0.07	-1.07	0.06	-4.08	*MT-ND1*	YP_003024026.1
CHH	9727	+	4.18E-06	8.12E-04	1.19	0.08	-1.11	0.06	-3.98	*MT-CO3*	YP_003024032.1
CHG	2419	+	2.34E-05	1.28E-03	0.75	0.05	-0.70	0.06	-3.95	*MT-RNR2*	_
CHG	3983	+	1.29E-03	1.58E-02	1.09	0.07	-1.02	0.07	-3.92	*MT-ND1*	YP_003024026.1
CHH	3179	-	2.34E-04	6.37E-03	0.77	0.05	-0.72	0.07	-3.90	*MT-RNR2*	_
CHG	870	+	4.00E-03	3.35E-02	0.78	0.05	-0.73	0.07	-3.85	*MT-RNR1*	_
CHH	5624	+	2.77E-04	6.89E-03	0.71	0.05	-0.66	0.07	-3.75	*MT-TA*	_
CHH	14875	+	1.72E-04	5.36E-03	0.04	0.53	0.49	13.24	3.73	*MT-CYB*	YP_003024038.1
CHG	11186	+	9.54E-05	2.80E-03	1.03	0.08	-0.95	0.08	-3.69	*MT-ND4*	YP_003024035.1
CpG	3162	+	1.53E-03	1.82E-02	0.85	0.07	-0.78	0.08	-3.63	*MT-RNR2*	_

+, heavy strand; -, light strand; rRNA, ribosomal RNA; tRNA, transfer RNA; [1] adjusted p-value, [2] mean methylation percentage, [3] difference in methylation percentages between patients and controls (patients - controls), [4] elements in the exact position of the methylation site.

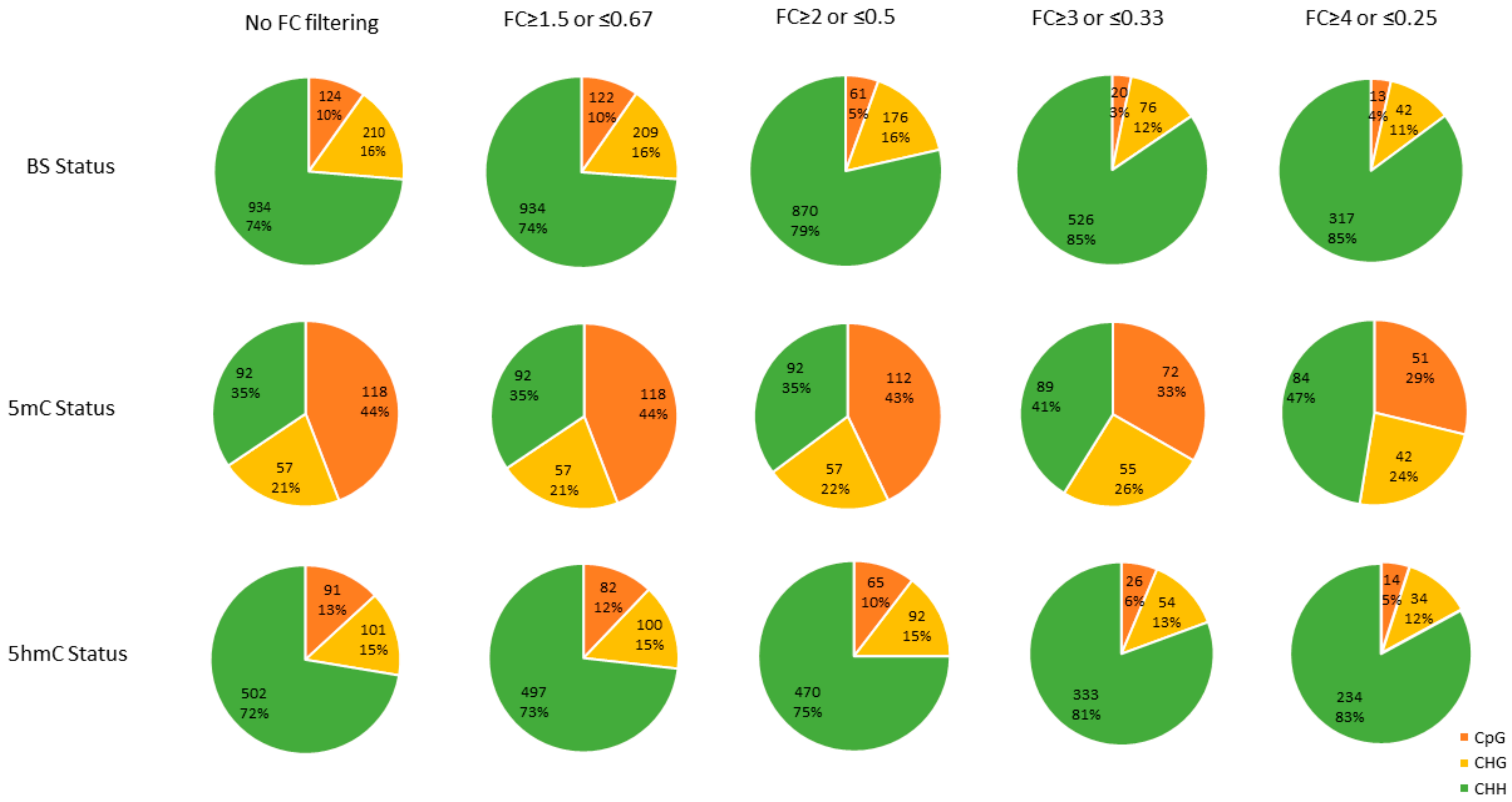

Supplementary Figure S1 | Pie charts illustrating the change in context representation of the differentially methylated cytosine (C) sites between EOPD patients and age- and sex-matched controls at different fold change (FC) filters. The differentially methylated C sites at ≥30X and q-value<0.05 are shown per context (CpG, CHG, and CHH). The CHH context representation is increased at more stringent FC filters, whereas the CpG context representation is decreased. This pattern is observed across all statuses (BS, 5mC, and 5hmC) at ≥30X. There is a maximum 1% change in context representation with no FC filtering compared with FC≥1.5 or ≤0.67 filter. However, at the FC≥4 or ≤0.25 filter, more than half of the differentially methylated C sites at the CpG context are lost. This observation is important when comparing results across different studies.

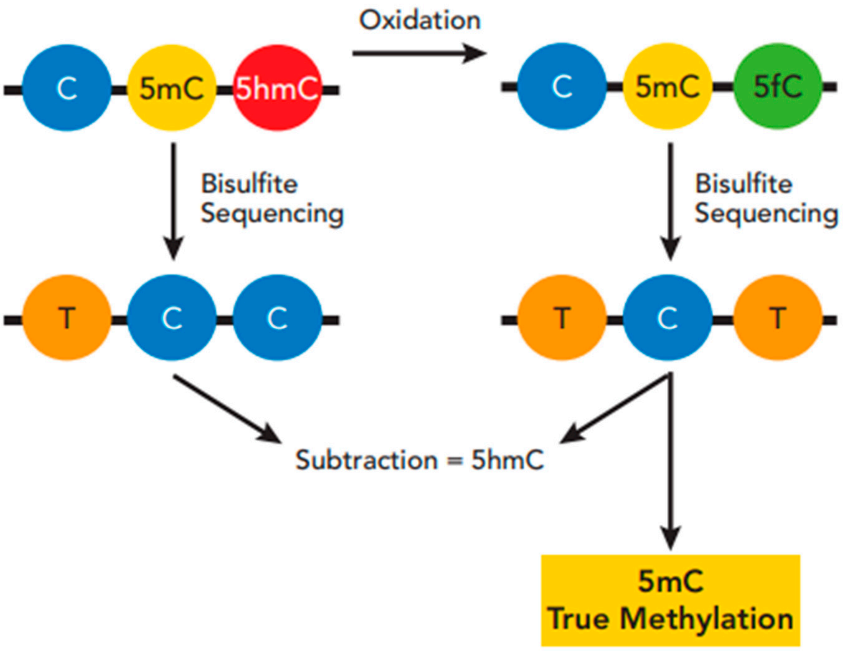

Supplementary Figure S2 │TrueMethyl oxBS module workflow. The TrueMethyl oxBS Module allows for the quantification of both 5-hydroxymethylcytosine (5hmC) and 5-methylcytosine (5mC). The site-specific oxidation step converts only 5hmC bases to 5-formylcytosine (5fC), without altering the 5mC bases. Bisulfite (BS) conversion converts the 5hmC and 5mC bases to cytosine (C), and the C and 5fC to thymine (T). When aliquots of the same sample are being processed in parallel through the oxidative (ox)BS and BS-only workflows, the content of 5hmC and 5mC can be determined through the subtractive analysis method. The oxBS workflow allows for the quantification of the C bases, which are representative of the true 5mC content, i.e. true methylation. Taken from the Ultralow Methyl-Seq with TrueMethyl® oxBS Protocol (M01512, v2, NuGEN® Technologies, USA).
